# Histologic Assessment of Drug-Eluting Grafts Related to Implantation Site

**DOI:** 10.3390/jdb4010011

**Published:** 2016-02-20

**Authors:** Jean-Christophe Tille, Sarra de Valence, Delia Mandracchia, Benjamin Nottelet, Francesco Innocente, Robert Gurny, Michael Möller, Beat H. Walpoth

**Affiliations:** 1 Division of Clinical Pathology, University Hospital of Geneva, Geneva 1211, Switzerland; 2 School of Pharmaceutical Sciences, University of Geneva, Geneva 1211, Switzerland; sarra.devalence@gmail.com (S.d.V.); benjamin.nottelet@univ-montp1.fr (B.N.); Robert.Gurny@unige.ch (R.G.); Michael.Moeller@unige.ch (M.M.); 3 Department of Pharmacy-Drug Sciences, University of Bari “Aldo Moro“, Bari 70125, Italy; delia.mandracchia@uniba.it; 4 Department of Cardiovascular Surgery, Faculty of Medicine, University Hospital, Geneva 1211, Switzerland; ufficiostampa@hpg23.it (F.I.); Beat.Walpoth@hcuge.ch (B.H.W.)

**Keywords:** vascular prosthesis, tissue engineering, extracellular matrix, drug release, biodegradable polymers, foreign body reaction, pathology

## Abstract

Drug-eluting vascular prostheses represent a new direction in vascular surgery to reduce early thrombosis and late intimal hyperplasia for small calibre grafts. Subcutaneous implantation in rats is a rapid and cost-effective screening model to assess the drug-elution effect and could, to some extent, be useful to forecast results for vascular prostheses. We compared biological and histological responses to scaffolds in different implantation sites. Polycaprolactone (PCL), paclitaxel-loaded PCL (PCL-PTX) and dexamethasone-loaded PCL (PCL-DXM) electrospun scaffolds were implanted subcutaneously and in an infrarenal abdominal aortic model in rats for up to 12 weeks. At the conclusion of the study, a histological analysis was performed. Cellular graft invasion revealed differences in the progression of cellular infiltration between PCL-PTX and PCL/PCL-DXM groups in both models. Cell infiltration increased over time in the aortic model compared to the subcutaneous model for all groups. Cell counting revealed major differences in fibroblast, macrophage and giant cell graft colonisation in all groups and models over time. Macrophages and giant cells increased in the PCL aortic model; whereas in the subcutaneous model these cell types increased only after three weeks or even decreased in the drug-eluting PCL groups. Other major findings were observed only in the aortic replacement such as extracellular matrix deposition and neo-angiogenesis. The subcutaneous implant model can be used for screening, especially when drug-eluting effects are studied. However, major histological differences were observed in cell type reaction and depth of cell penetration compared to the aortic model. Our results demonstrate that the implantation site is a critical determinant of the biological response.

## 1. Introduction

Biomaterial implants are used in a variety of anatomic locations. It has been recognized that maintaining a controlled biological reaction in the peri-implant region is essential. Although final device designs are mostly used in their target anatomical location, the biocompatibility of polymer components is often evaluated subcutaneously or intramuscularly.

The rat subcutaneous tissue implant site has proven to be a high-throughput, relatively low-cost screening technique for testing tissue responses to new materials [1,2]. The healing characteristics around polymer implants in subcutaneous tissues are often extrapolated to other tissues with the expectation that the observed healing will be similar in other locations.

This model could also allow an initial evaluation of the effects of a drug-loaded graft on tissue responses in a more relevant environment than the *in vitro* assay.

Biostable synthetic grafts such as expanded polytetrafluorethylene (ePTFE) have different healing and cellular infiltration characteristics in different implantation sites, e.g., subcutaneous *versus* adipose tissue in the rat [3,4]. Similarly, ePTFE grafts implanted in the abdominal wall showed different cell colonization in the prosthesis compared to vascular implantation [5].

Drug incorporation in fibers using electrospinning of drug-loaded solutions has been described for various compounds and is a promising approach for controlled drug delivery applications related to tissue engineering [6,7].

The aim of this study was to prepare drug-eluting biodegradable grafts with a defined and comparable nanostructure, and perform an *in vivo* assessment in two different anatomical locations, one with a pulsatile flow and the other without, in order to compare the drug-effect and cellular response.

## 2. Results

### 2.1. In Vitro Studies

#### 2.1.1. Fibre Morphology and Mechanical Properties

**Figure 1:**
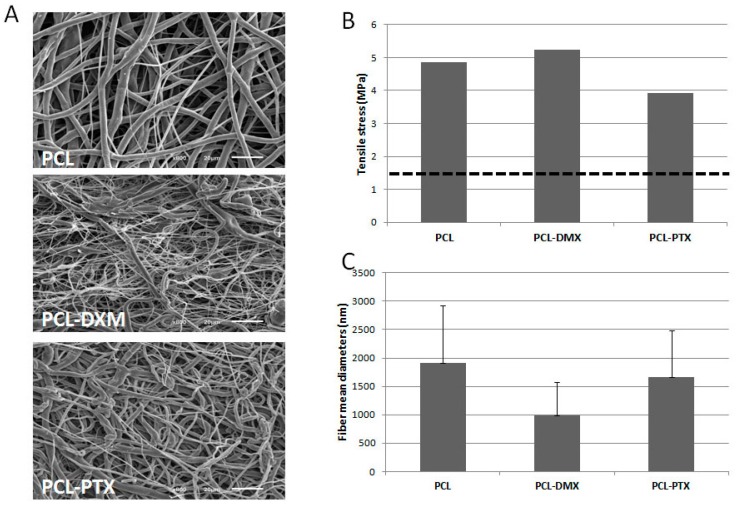
Morphological and mechanical properties of biodegradable polycaprolactone (PCL) electrospun grafts. (**A**) SEM pictures of graft inner surfaces at 700× magnification for PCL (12% *wt*/*vol*), PCL (9% *wt*/*vol*)-DXM (1%) and PCL(12% *wt*/*vol*)-PTX (0.75%); (**B**) Tensile stress values of PCL (12% *wt*/*vol*), PCL (9% *wt*/*vol*)-DXM (1%) and PCL (12% *wt*/*vol*)-PTX (0.75%) gamma sterilized graft (*n* = 1 for each); dott line correspond to “native” vessels tensile stress of 1.4 MPa; (**C**) Mean fiber diameters of PCL (12% *wt*/*vol*), PCL (9% *wt*/*vol*)-DXM 1% and PCL (12% *wt*/*vol*)-PTX (0.75%) gamma sterilized graft. (Data are expressed as mean ± SD and correspond to fiber diameter mean values calculated from 100 measurements).

A mechanical and morphological evaluation of the PCL-DXM compared to PCL-PTX and non-loaded PCL grafts was carried out. For the same PCL concentration during electrospinning, DXM-loaded grafts have higher maximal stress and strain values than non-loaded grafts (data not shown), as previously reported for PTX-loaded grafts [8]. Fiber diameter (Figure 1A,C) and tensile strength (Figure 1B)were comparable for PCL and PCL-PTX scaffolds. In terms of morphology and as illustrated in Figure 1A, PCL-DMX loaded fibers of the surface present an average smaller diameter but still are in the same range as the PCL and PCL-PTX ones (*i.e.*, around 1 µm, Figure 1C). This difference had little impact on tensile stress as it was of *ca.* 5 MPa for PCL-DXM against *ca.* 4 MPa for PCL-PTX and 5 MPa for PCL. All these values are above the value of 1.4 MPa found for natural vessels.

#### 2.1.2. Evaluation of DXM-Loading and Incorporation Efficiency

Studies were carried out to quantify DXM loading into the graft. PCL-DXM grafts loaded with 1% DXM (*wt*/*wt*) yielded a high incorporation ranging from 64% to 98%, representing 130–206 mg DXM for a 2.5 cm long graft. The drug incorporation data for the PCL-PTX graft have been reported previously [8].

#### 2.1.3. *In Vitro* DXM Release Profile

**Figure 2:**
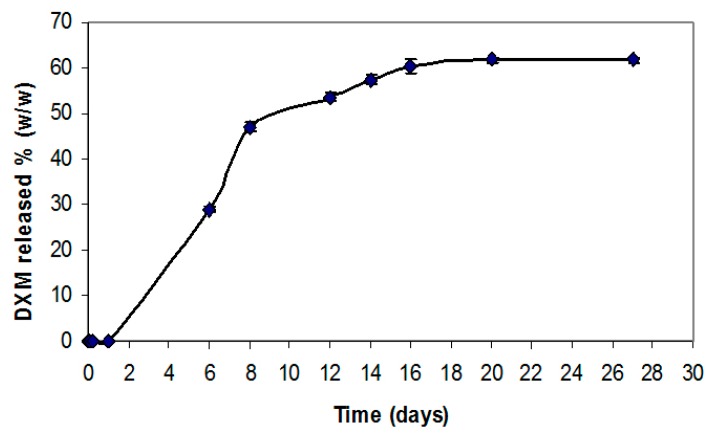
*In vitro* drug release profiles of a PCL graft containing 1% (*wt*/*wt*) DXM over 16 days. Data are expressed as an average of results obtained from 3 grafts at each time point (mean ± SD).

The results of DXM release from the electrospun PCL graft *in vitro* show an initial time lag on the first day, probably related to the high interaction of the hydrophobic drug with the polymer matrix. At day 6, approximately 30% of the drug had been released followed by a slower release phase over the following 16 days of 60% of the initially incorporated drug (Figure 2). The release profiles from the PCL-PTX graft have been previously reported [8].

### 2.2. In Vivo Studies

#### 2.2.1. *In Vivo* Rat Subcutaneous Model

Unloaded-PCL, PCL-PTX (0.5% and 0.75%) or PCL-DXM (1%) grafts were implanted subcutaneously in rats for 1, 3 and 12 weeks to determine the *in vivo* anti-proliferative effect for PTX and anti-inflammatory effect for DXM.

**Figure 3:**
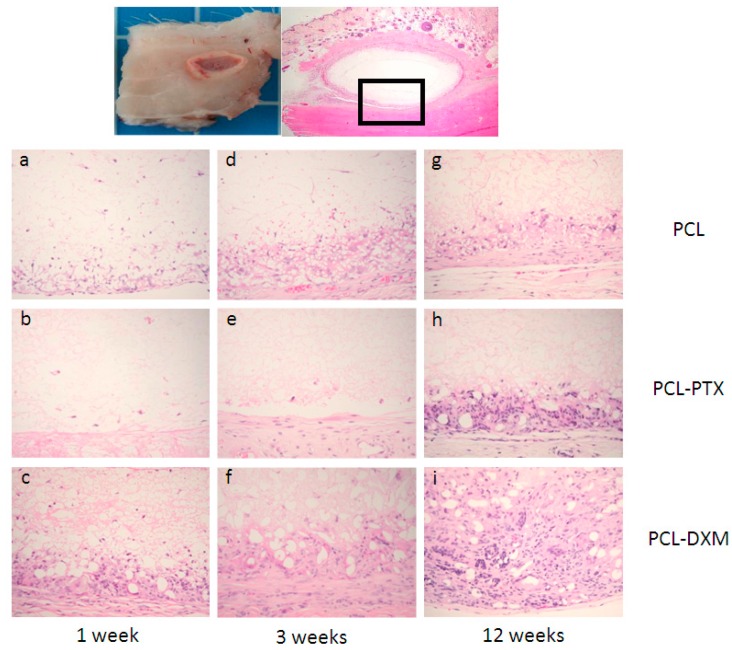
Histological analysis of the subcutaneous implant model. Subcutaneous implants and HE staining. The squares in the first row indicate the localisation of the magnifications below. Implant at 1 week (**a**–**c**), 3 weeks (**d**–**e**) and 12 weeks (**g**–**i**) of PCL grafts (**a**,**d**,**e**), PCL-PTX 0.75% (**b**,**e**,**h**) and PCL-DXM (**c**,**f**,**i**). HE staining, magnification 200×. The luminal side is on the top of the pictures.

After 1 week of implantation, a major difference between PCL and PCL-PTX was observed. A full histological description of PCL grafts has been previously reported in the aorta [9]. Compared to PCL, PCL-PTX grafts had neither a peri-graft cellular reaction, a macrophage or giant cell reaction, nor cellular infiltration within the grafts (Figure 3a,b). The effect was similar for PCL-PTX-loaded grafts at 0.5% and 0.75% and both groups were combined for further evaluation. On the other hand, PCL-DXM grafts had stronger giant cell and macrophagic reactions compared to PCL (Figure 3c).

Between 1 and 3 weeks after implantation, the cellular infiltration of PCL and PCL-DXM grafts increased with higher numbers of giant cells, macrophages and some fibroblasts (Figure 3d,f). No cellular reaction was observed in the PCL-PTX group (Figure 3e).

After 12 weeks of implantation, all grafts were colonized by host cells from the external part with a giant cell reaction at the periphery of the graft to the internal part with macrophages and some fibroblasts inside the graft wall. At the 12 week time point, no difference could be observed between PCL and PCL-PTX grafts (Figure 3g,h). In PCL-DXM grafts, the giant cells and macrophagic reaction was stronger compared to PCL and PCL-PTX grafts (Figure 3i).

**Figure 4:**
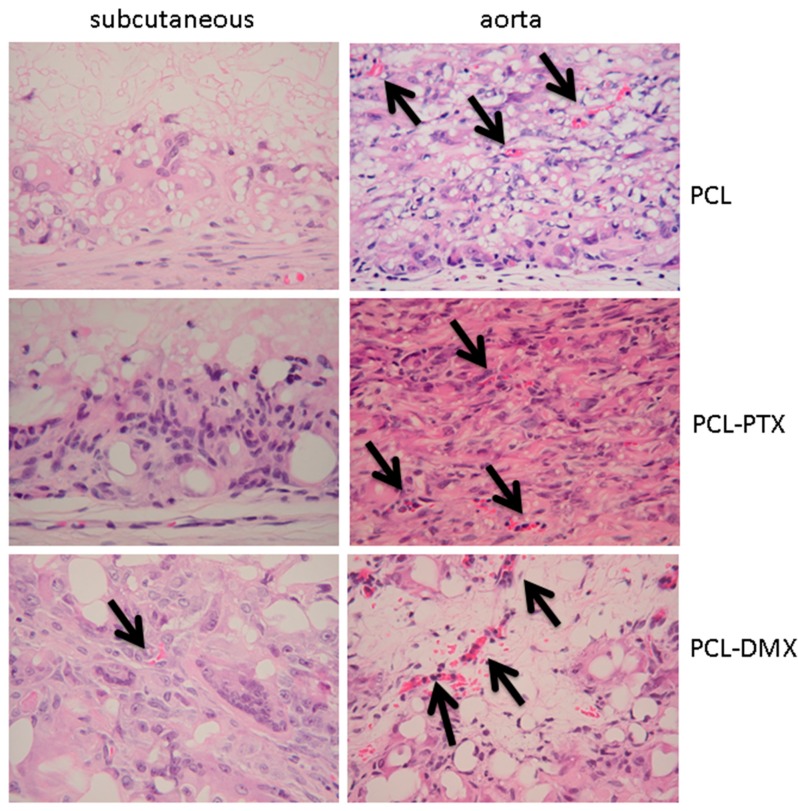
Histological analysis of neoangiogenesis in both models. Implants at 12 weeks in the subcutaneous and aortic models. Arrows indicate capillaries. HE staining, magnification 400×.

No calcification was observed within the grafts in all groups and at all time-points. A few capillaries (neo-angiogenesis), were found focallyin one 12-week PLC-DXM graft at 12 weeks, but this was not seen in the PCL or PCL-PTX grafts at any of the time points (Figure 4).

#### 2.2.2. *In Vivo* Rat Aorta Interposition Model

We compared *in vivo* aorta implantation studies [8,9] to subcutaneous implantations to first compare the effect of the drugs and, second, the cellular reaction in and around the graft in different *in vivo* implantation sites.

**Figure 5:**
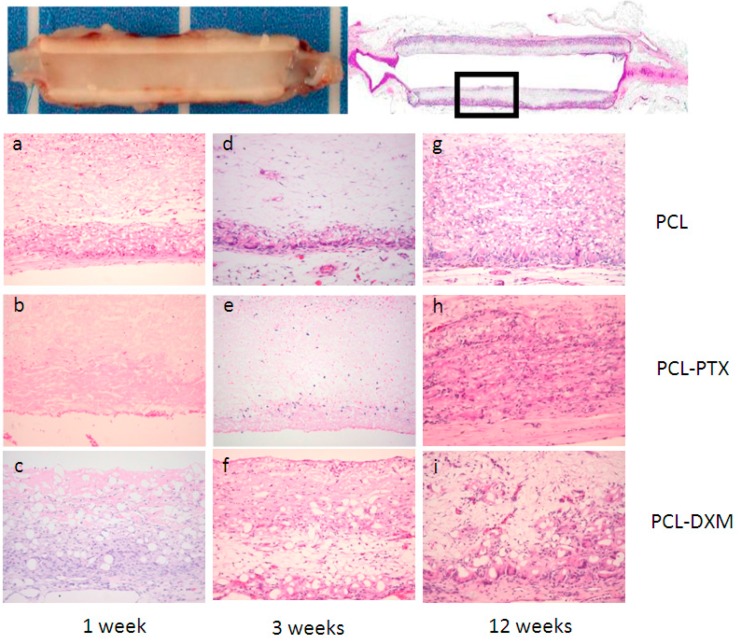
Histological analysis of the aorta graft model. Upper part: representative images of an aortic graft implant, the square indicates the location of the magnifications below. Vascular implant at 1 week (**a**–**c**), 3 weeks (**d**–**e**) and 12 weeks (**g**–**i**) of PCL graft (**a**,**d**,**e**), PCL-PTX 0.75% (**b**,**e**,**h**) and PCL-DXM (**c**,**f**,**i**). HE staining, magnification 200×. The luminal side is on the top.

One week after implantation, PCL-PTX explanted aortic grafts had no cellular reactions or infiltration as observed in the subcutaneous model (Figure 5b). The PCL-DXM group demonstrated a stronger cellular reaction with fibroblast infiltration and macrophages compared to the PCL group (Figure 5a,c). Compared to the subcutaneous implant model, the cellular reaction present in the PCL-DXM vascular group appeared to be more pronounced.

After 3 weeks of implantation, some fibroblasts infiltrated the PCL-PTX grafts with no giant cells or macrophages (Figure 5e). PCL-DXM and PCL groups showed the same aspect with giant cells formation, macrophages and some fibroblasts in the graft (Figure 5d,f).

After 12 weeks of implantation, PCL-PTX, PCL-DXM and PCL grafts had identical cellular reactions, which were composed of giant cells, macrophages and fibroblasts. The macrophagic reaction was lighter in PCL-DXM grafts compared to others grafts (Figure 5g–i).

Neoangiogenesis was observed at 12 weeks in the grafts for all groups (Figure 4). Calcifications in the intima hyperplasia were present in all groups at 12 weeks near the anastomosis or in the middle of the graft and developed through chrondroid metaplasia (data not shown), as previously reported [9].

#### 2.2.3. Morphological Cellular Invasion of the Grafts

Cell type infiltrations, giant cells, macrophages and fibroblasts within the grafts were counted in the subcutaneous and vascular models.

**Figure 6:**
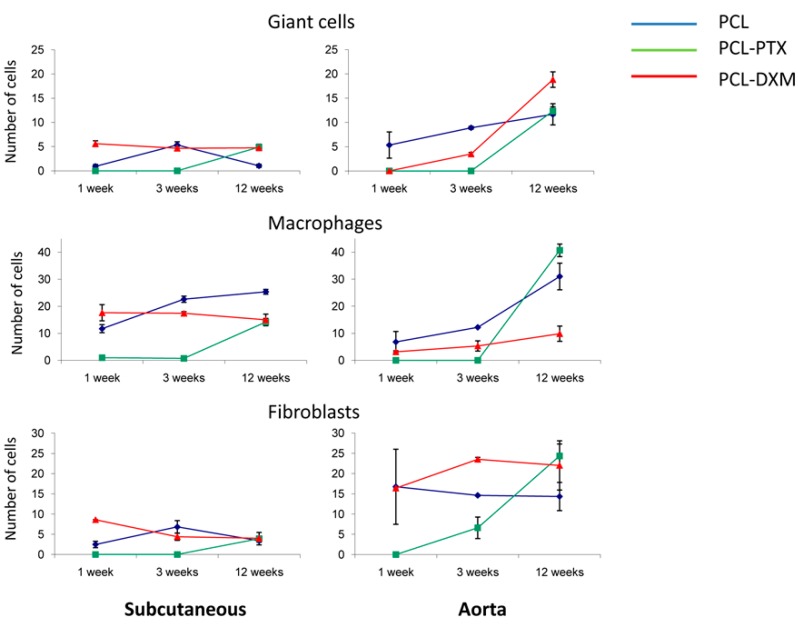
Type of cellular infiltration. The subcutaneous model (left column) and the aortic model (right column). Data are expressed as an average count of various cell types from all grafts at all time points with SEM.

The giant cell reaction was stable in PCL-DXM grafts in the subcutaneous model and had a transient increase from 1 to 3 weeks in the PCL graft. PCL-PTX grafts had an increase in giant cells at 12 weeks (Figure 6, top left). In the aortic location, the number of giant cells increased continuously from 1 to 12 weeks for PCL and PCL-DXM and appeared at 12 weeks for PCL-PTX, similarly to the subcutaneous model (Figure 6, top right). Giant cells numbers at 1 week were higher in the subcutaneous model for PCL-DXM compared to the aortic model and lower in the PLC graft in the subcutaneous model at 1 week compared to the aortic model.

The cellular density of macrophages remained stable in the PCL-DXM graft in the subcutaneous model and increased over time in PCL and PCL-PTX graft (Figure 6, middle left). In the aortic model, the number of macrophages increased over time in all three groups (Figure 6, middle right). Interestingly, the macrophage numbers in PCL and PCL-DXM grafts were significantly higher in the subcutaneous model at 1 and 3 weeks compared to the aortic model (*p* < 0.05).

Fibroblast numbers in the subcutaneous model had the same kinetic as the giant cells for PCL, PCL-DXM and PCL-PTX grafts (Figure 6, lower left). In the aortic model, fibroblast numbers slightly decreased in PCL grafts and remain stable in PCL-DXM grafts from 3 to 12 weeks. In PCL-PTX grafts some fibroblasts infiltrated the graft at 3 weeks and increased up to 12 weeks (Figure 6, lower right). Compared to the subcutaneous model, the fibroblast numbers were higher in the aortic model for the three grafts.

#### 2.2.4. Morphometric Analysis

Quantitative morphometric cellular graft infiltration between PCL, PCL-PTX and PCL-DXM grafts in the subcutaneous and aorta interposition models was compared over time.

**Figure 7:**
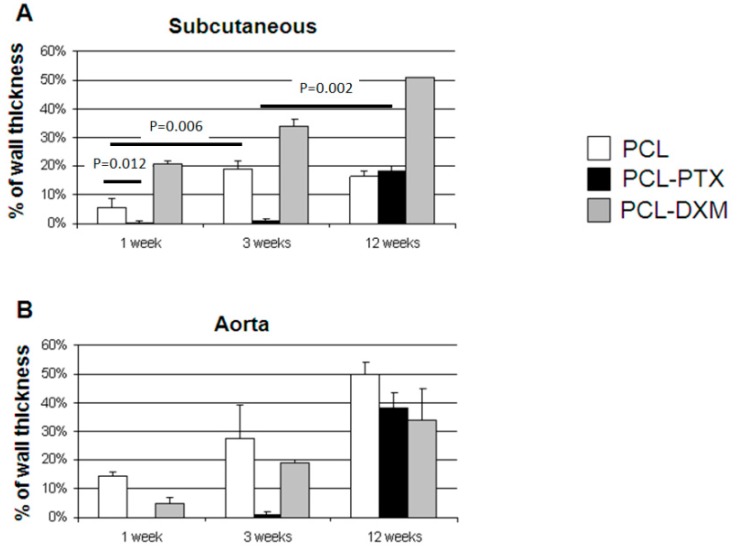
Morphometric cellular infiltration of the grafts. Percentage of cellular ingrowth into the grafts at 1, 3 and 12 weeks in the subcutaneous model (**A**) and the aortic model (**B**) (mean values ± SEM).

In the subcutaneous model, the cellular invasion in PCL grafts increased up to 3 weeks (5.5% at 1 week and 18.8% at 3 weeks) and remained stable up to 12 weeks (16.5%) (Figure 7A). In the PCL-DXM grafts cellular invasion continuously increased from 1 to 12 weeks after implantation (20.9% at 1 week, 33.8% at 3 weeks and 51.1% at 12 weeks) (Figure 7A). The anti-proliferative effect of PTX on cellular infiltration was active at 1 and 3 weeks, then vanished at 12 weeks, with a cellular infiltration similar to PCL grafts (18.2% at 12 weeks) (Figure 7A).

In the aortic model, the cellular invasion of the graft increased continuously over time from 1 to 12 weeks in all grafts (Figure 7B). The delay of the cellular graft infiltration observed for the subcutaneous PCL-PTX graft was also present in the aortic model. An interesting point was that the percentage of cellular infiltration was higher in the aortic model for PCL and PCL-PTX graft and lower in the aortic model for PCL-DXM compared to the subcutaneous model.

#### 2.2.5. Extracellular Matrix Formation

**Figure 8:**
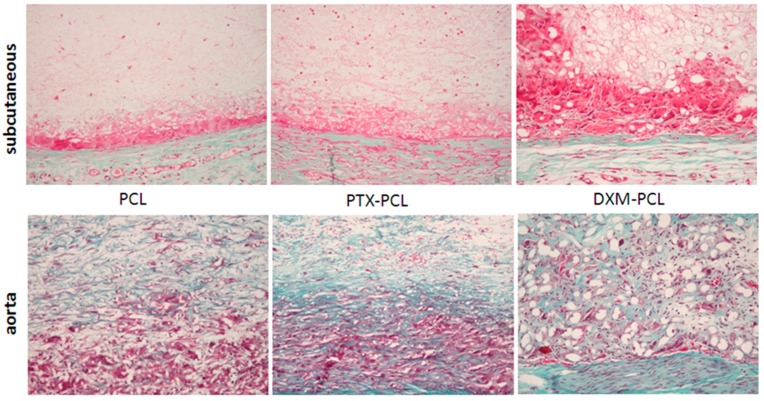
Collagen deposition in the graft. Miller-Masson combined staining of the subcutaneous model (upper panel) and the aortic model (lower panel) at 12 weeks. No collagen deposition (green) was found in the subcutaneous model. Magnification 200×.

In order to compare the collagen synthesis and deposition in the graft, Miller-Masson staining was performed at all time points in the three different grafts. In the subcutaneous model, there was no collagen deposition from 3 to 12 weeks (Figure 8). On the other hand, in the aortic model, collagen deposition in the graft was present from 6 weeks and increased at 12 weeks in all grafts (Figure 8).

## 3. Discussion

This study served as a comparative evaluation for the drug-eluting biodegradable graft effect and host cellular reaction *in vivo* in a classical subcutaneous model compared to an aortic replacement model used for small vascular grafts. Historically, several extrapolations from subcutaneous implant models had been made when testing new materials for clinical applications, but direct comparisons between anatomical implantation sites have rarely been published.

A detailed assessment of the prepared and investigated PCL, PCL-PTC and PCL-DXM, respectively, proved their comparability in their mechanical and morphological properties, as well as their drug release characteristics.

We observed a marked biological effect of PTX in both subcutaneous and aortic models with a delayed cellular reaction and graft invasion. DXM does not seem to affect the host reaction in a time dependent manner compared to unloaded-PCL, but a stronger giant cell reaction was observed in the subcutaneous model compared to the aortic model. This could be due to the slight differences in fibre surface nanostructures and alignments. The macrophagic reaction was not lower in PCL-DXM grafts, possibly due to the low DXM dosage, below 0.5 mg/kg. Another explanation is that DXM, a well-known immune modulator, had no effect on bone marrow-derived macrophages’ differentiation *in vitro* and also decreased macrophages’ adhesiveness [10,11]. This mechanism could also be taken into account for the increased macrophage infiltration.

Cellular infiltration of the grafts over time was the main difference between the two *in vivo* models. In the subcutaneous model, the cellular infiltration increased for PCL-PTX and PCL-DXM up to 12 weeks and was stable for three weeks for PCL. Interpretation at the one week time point is limited since only one PCL-PTX graft was retrieved at one week. In contrast, in the aortic model, there was a continuously increased cellular infiltration in all three biodegradable grafts. We did not observe differences in the diameter or orientation of the fibres, indicating that these were not modified during the processing. We know that the scaffold nanostructure architecture is an important point with regard to the healing process and host response *in vivo* [12]. This difference could possibly be related to the vascular environment with a blood flow induced mechanical strain on the grafts promoting scaffold structure modification and cell mobilization within the graft, as shown for ePTFE implants [5].

The magnitude of the macrophage and fibroblast reaction to the graft was also different for the subcutaneous and the aortic models. The numbers of fibroblasts in the grafts were higher in the subcutaneous model and the opposite was seen for macrophages, which infiltrated in lower numbers at early time points in the subcutaneous model. This difference in cell type reaction and infiltration could be explained by the differences between the models: a static subcutaneous model *vs.* a pulsatile aortic model. Extracellular matrix formation in the graft, such as collagen—a key component for mechanical stability—was only present in the aortic model and not observed in the subcutaneous model. This difference in extracellular accumulation was also observed by others in bio-reactor matured models of tissue-engineered vessels. Grafts in a pulsatile *in vitro* flow system contained more matrix deposition compared to static conditions, indicating an effect of pulse on fibroblasts [13,14].

Calcification located only in the intima hyperplasia was only present in the aortic model at 12 weeks in all grafts and never in the subcutaneous model. This may also be linked to the pulsatile conditions.

Neoangiogenesis, accompanying cellular colonization of the graft, is an important factor for tissue remodelling. Neoangiogenesis was observed only focally in one subcutaneously implanted PCL-DXM graft, but was present in all grafts implanted in the aortic model after 12 weeks.

## 4. Materials and Methods

### 4.1. Preparation of Unloaded, PTX-Loaded and DXM-Loaded PCL Grafts by Electrospinning

Two mm inner diameter unloaded PCL and paclitaxel-loaded PCL (PCL-PTX, with two different PTX concentration: 0.5% and 0.75%) grafts were prepared according to a process optimized by a factorial design approach that has been reported in detail elsewhere [8,9,15].

In a similar factorial design approach, various optimized dexamethasone-loaded grafts (PCL-DXM) were prepared, and the effects of different DXM concentrations on fibre morphology, graft tensile stress and strain behaviours were investigated. Briefly, the DXM-loaded grafts were prepared in the same manner as the unloaded controls by electrospinning of PCL solutions (7.5%, 9% and 12% [*wt*/*vol*]) containing different amounts of DXM (1% and 10% [*wt*/*wt*]). The generated fibres were collected on a rotating (4500 rpm) and translating (200 rpm, 4-cm amplitude) mandrel to form a nonwoven tubular PCL graft. The grafts were dried at 37 °C under vacuum overnight. Residual solvents were analysed with a Headspace Sampler (HP7694, Hewlett-Packard, Palo Alto, CA, USA) coupled to a gas-chromatography system (GC System, 6850 Series, Agilent Technologies, Santa Clara, CA, USA) equipped with an HP-1 capillary column (Agilent Technologies). All grafts were γ-sterilized at 25 kGy before characterization and *in vivo* use. The PCL-DXM graft chosen for *in vivo* studies was made from a PCL 9% [*wt*/*vol*] PCL solution with DXM 1% because it showed similar fibre morphology and mechanical properties to plain PCL grafts used previously [8,9,14].

### 4.2. Assessment of DXM-Loading and Distribution in the Graft

The extent of drug loading and the incorporation efficiency were assessed by high performance liquid chromatography (HPLC, Acquity UPLC system, Waters Corporation, Milford, CT, USA) analysis in 2.5 cm long DXM-loaded grafts with a theoretical loading of 1% and 10% [*wt*/*wt*]. Additionally, the homogeneity of DXM distribution along the graft (*n* = 3) was verified by cutting four 1 cm long pieces from a 4 cm DXM-loaded graft.

### 4.3. Assessment of DXM Release

Grafts theoretically loaded with 1% of DXM (*n* = 3) were placed in vials with an exact measured amount of release medium of phosphate buffered saline (pH = 7.4) containing 0.05% (*vol*/*vol*) Tween 20. The vials were kept at 37 °C with rotational mixing at 100 rpm for 1, 5, and 16 h and 1, 6, 8, 12, 14 and 16 days. At each time point, 1 mL aliquots of the release medium were taken for DXM quantification. The remaining release medium was aspirated and fresh release medium was added at each time point to maintain sink conditions.

### 4.4. Mechanical and Morphological Properties Evaluation

Fibre diameter and morphology of the grafts were investigated by scanning electronic microscopy (SEM, JEOL JSM-6510LV, Tokyo, Japan) as previously reported [8,9,15]. Briefly, tensile stress was measured on complete vascular grafts using a mechanical testing bench from Schenck AG (Nänikon, Dübendorf, Switzerland) using a cross-head speed of 10 mm/min at room temperature. The vascular grafts were fixed by clamps using 5 mm of each end of the graft, so that either a 1 or 3 cm length was used for the measurements corresponding for the 2 and 4 mm, respectively, I.D. grafts.

#### Statistical Analyses

A statistical analysis was performed only for the cellular invasion in the subcutaneous model with a Mann Whitney U test two-tailed (http://www.socscistatistics.com). Due to a small number of samples, it was not possible to perform a statistical analysis for the PCL-DXM sample in this model and also in the aorta model for all samples. A *p* < 0.05 was considered as significant.

### 4.5. In Vivo Studies

The experimental protocol was approved by the Animal Experiments Ethical Committee of the University of Geneva (Protocol No: 06/52) and the Veterinary Office of State of Geneva, Switzerland (No: 1081/3232/II), and carried out in conformity with the Guide for Care and Use of the Laboratory Animals (National Research Council, Washington, DC: National Academy Press; 1996).

#### 4.5.1. Rat Subcutaneous Model

*In vivo* studies were carried out on 60 male Sprague–Dawley rats (375 g) according to the ethical principles of laboratory animal care and Swiss regulations. The animals were kept in individual cages and received food and water *ad libitum*. Anaesthesia was induced with isoflurane 4% and the animals were maintained under deep anesthesia with isoflurane 2%, 40% oxygen through facial masks.

After shaving the abdominal region, we implanted PCL, PCL-PTX, PCL-DXM grafts (10 mm length and 2 mm internal diameter, wall thickness 600 µm) subcutaneously with a maximum of 6 grafts per rat. The animals were followed for 1 week (PCL *n* = 8, PCL-PTX *n* = 10 (0.5% = 2, 0.75% = 8), PCL-DXM *n* = 3), 3 weeks (PCL *n* = 9, PCL-PTX *n* = 12 (0.5% = 3, 0.75% = 9), PCL-DXM *n* = 3) and 12 weeks (PCL *n* = 6, PCL-PTX *n*=6 (0.5% = 2, 0.75% = 4), PCL-DXM *n* = 3). Euthanasia was then performed under deep isoflurane anesthesia by intracardiac injection of KCl. At sacrifice only 2 PCL-DXM and 1 PCL-DXM grafts were found remaining at the implantation site at 1 and 12 weeks, respectively. All other grafts were retrieved.

#### 4.5.2. Rat Aorta Interposition Model

Our experimental rat model, including the anaesthesia and operative techniques as well as the follow-up protocol, was described in detail previously [9]. Briefly, 28 male Sprague-Dawley rats (375 g) were anaesthetized and operated on according to this protocol. All grafts (10 mm length and 2 mm internal diameter, wall thickness 600 µm) were implanted randomly into the rat abdominal aorta. All rats were followed for 1 week (PCL *n* = 3, PCL-PTX *n* = 3, PCL-DXM *n* = 3), 3 weeks (PCL *n* = 3, PCL-PTX *n* = 3, PCL-DXM *n* = 3) and 12 weeks (PCL *n* = 3, PCL-PTX *n* = 3, PCL-DXM *n* = 3). No anticoagulation or anti-platelet drugs were given. At conclusion of the study period, digital subtraction angiography was performed to assess patency and rats were sacrificed after explantation of the vascular graft with the adjacent abdominal aorta segments. All grafts were patents at all time points.

#### 4.5.3. Histological Assessment and Quantitative Morphometric Analysis

For subcutaneous implants, the area of implantation was dissected and fixed in 4% formaldehyde for 24 h. A slice of the subcutaneously implanted graft with adjacent tissue was embedded in paraffin wax. For the aorta, explanted grafts with both anastomoses were fixed in 4% formaldehyde for 24 h, cut into two longitudinal halves, and then embedded into paraffin. Histological sections of 4 μm were stained with Hematoxylin-Eosin (HE) and Miller-Masson for elastin and collagen deposition.

#### 4.5.4. Morphological Cell Quantification Analysis

**Figure 9:**
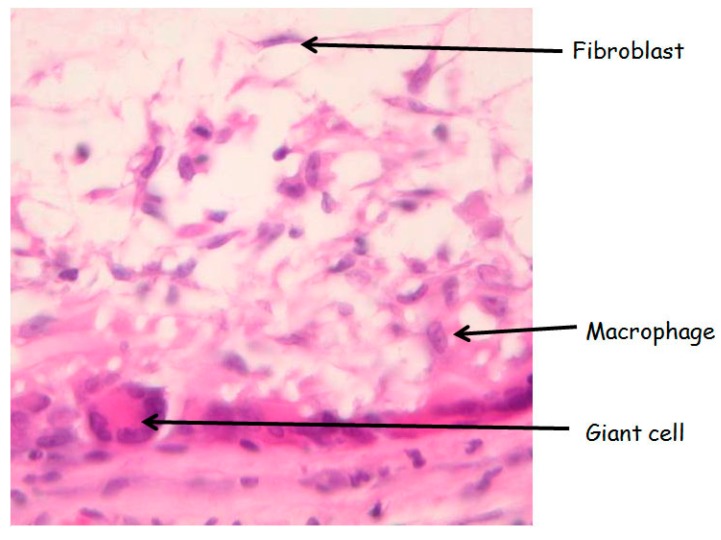
Morphology of the different analysezed cells type. HE stain (magnification 400×).

In the subcutaneous and vascular explants, based on classical morphology, giant cells around the graft, macrophages and fibroblasts within the graft were counted under a light microscope at 400× magnifications over 5 representative fields on HE stains, and the final cell number was per 1 high power field (400× magnification). Morphological identification of the different cells types was done with the following criteria: macrophages were identified by an abundant cytoplasm with an eccentric reniform nucleus; giant cells are large cell with numerous nuclei; fibroblasts have a spindle-shape with an elongated nucleus and little cytoplasm, as illustrated below (Figure 9).

Neoangiogenesis was analyzed on HE stains and capillaries were defined as a small vessel circled by endothelial cells and containing erythrocytes in the lumen. Slides were digitalized with the Mirax scan (Carl Zeiss MicroImaging GmbH, Göttingen, Germany) and images analyzed with ImageJ software (version 1.3, National Institute of Health, Bethesda, MD, USA). Cellular invasion of the graft was defined as the percentage of graft area which was penetrated densely by host cells from the external side towards the inner side.

## 5. Conclusions

The subcutaneous implant model for testing biodegradable polymers can be used for screening, especially when drug-eluting effects are studied. However, major histological differences in cell reaction, such as the depth of cell colonization compared to the aortic replacement model, indicate some limitations and demonstrate that the results from one model cannot be generalized for later application at a different implantation site. It is therefore highly recommended to perform the implantation at the site of interest in order to confirm initial results obtained in the subcutaneous implant model, especially for drug releasing, biodegradable implants.
